# Effect of TiB Orientation on Near-Threshold Fatigue Crack Propagation in TiB-Reinforced Ti-3Al-2.5V Matrix Composites Treated with Heat Extrusion

**DOI:** 10.3390/ma12223685

**Published:** 2019-11-08

**Authors:** Shoichi Kikuchi, Shunsuke Tamai, Takao Kawai, Yoshikazu Nakai, Hiroki Kurita, Sophie Gourdet

**Affiliations:** 1 Department of Mechanical Engineering, Faculty of Engineering, Shizuoka University, Shizuoka 422-8017, Japan; 2 Department of Mechanical Engineering, Kobe University, Kobe, Hyogo 657-8501, Japan; 1534552t@stu.kobe-u.ac.jp (S.T.); 171t318t@stu.kobe-u.ac.jp (T.K.); nakai@mech.kobe-u.ac.jp (Y.N.); 3 Department of Materials Processing, Graduate School of Engineering, Tohoku University, Miyagi 980-8577, Japan; kurita@material.tohoku.ac.jp; 4 Ariane Group, 78130 Les Mureaux, France; sophie.gourdet@ariane.group

**Keywords:** fatigue, fracture mechanics, titanium alloy, titanium boride, crack closure

## Abstract

TiB-reinforced Ti-3Al-2.5V matrix composites, in which TiB whiskers are oriented parallel to the direction of heat extrusion, were fabricated via mechanical alloying and hot isostatic pressing (HIP). To investigate the near-threshold fatigue crack propagation in TiB-reinforced Ti-3Al-2.5V matrix composites, stress intensity factor *K*-decreasing tests were conducted for disk-shaped compact specimens having two different orientations of TiB whiskers at force ratios from 0.1 to 0.8 under ambient conditions. The crack growth rates, d*a*/d*N*, for the composites incorporating TiB whiskers oriented perpendicular to the direction of crack growth were constantly lower than those obtained in the case where the orientation was parallel at the same stress intensity range Δ*K*, while the threshold stress intensity range, Δ*K*_th_, was higher. This effect can be explained by the increase in the degree of roughness-induced crack closure resulting from the perpendicular TiB, because fatigue cracks preferentially propagated across the boundaries between the matrix and the TiB in certain regions. In contrast, the effective threshold stress intensity range, Δ*K*_eff,th_, for composites was unaffected by the TiB orientation at low force ratios.

## 1. Introduction

As a result of their high heat resistance and superior specific strength, titanium alloys are frequently employed in aerospace components. Recently, there has been increasing interest in improving the structural reliability of such alloys by enhancing their mechanical properties, as a means of addressing the weight saving and downsizing for aerospace components. The microstructure of a metal typically determines its mechanical properties, and so modifying this microstructure could effectively improve the characteristics of materials based on titanium. Such modifications can be achieved using a variety of methods, including the generation of surface topography [1], the insertion of additional elements in an alloy [2,3,4,5,6,7,8,9,10,11], and grain refinement [11,12,13,14].

Prior work has demonstrated that the mechanical properties of stainless steels [15] and of alloys of magnesium [16], cobalt–chromium–molybdenum [17], and titanium [18,19,20,21,22,23,24,25,26,27,28,29,30] can all be improved via the addition of boron. The combination of titanium and boron generates whiskers of titanium boride (TiB) as a result of the reaction Ti + TiB_2_ → 2 TiB. Hyman et al. [31] and Li et al. [32] have reported that the growth of TiB occurred preferentially along the orthorhombic [010] direction during the sintering and hot isostatic pressing stages of composite fabrication. Feng et al. [33,34] characterized the microstructure of in situ synthesized TiB and interfacial structures, and clarified that the growth mechanism of TiB whiskers was a stacking process of the (100) plane of TiB. These whiskers exhibit chemical stability within the resulting alloys [25] because the coefficients of thermal expansion of pure titanium and of TiB are similar [26], such that residual thermal stress in the whiskers can be relieved within the alloy. Prior works [19,27,28] have shown that TiB whiskers provide titanium alloys with improved tensile strength, and so the incorporation of TiB is a potential means of reinforcing titanium matrix composites (TMCs). Furthermore, Wang et al. [30] has reported that both the tensile strength and elongation of the TiB-reinforced Ti matrix composites were sharply increased after extrusion, which suggests that the orientation of TiB influences the mechanical properties of Ti matrix composites. Schuh et al. [35] has examined the behavior of TiB whisker alignment during uniaxial superplastic elongation of the Ti-6Al-4V reinforced with 10 vol.% TiB whiskers.

The present work examined the fatigue resistance of titanium alloys reinforced with TiB treated with heat extrusion that show sufficient strength for practical engineering applications. The aim of this study was to establish the relationship between the orientation of TiB whiskers and crack propagation under near-threshold fatigue conditions in a Ti-3Al-2.5V alloy incorporating TiB. The mechanism by which fatigue cracks propagate through the alloy was also examined, based on fractography and the concept of crack closure.

## 2. Experimental Procedures

### 2.1. Materials

A powdered Ti-3Al-2.5V alloy (Crucible Materials Research, currently an Allegheny Technology, subsidiary, Pittsburgh, PA, USA,) having the composition provided in Table 1 was employed in this work. This material was generated by argon atomization and comprised spherical particles 58 µm in diameter. The TiB_2_ was a ceramic powder with a particle diameter of 4.8 µm and the composition provided in Table 2.

The experimental composite was obtained by combining the Ti-3Al-2.5V with 6.4 vol% TiB_2_, such that the mixture contained 10 vol% TiB. Details about the initial microstructure are described in the previous papers [36,37,38]. This was accomplished using a specialized high-energy mixing technique proprietary to Aerospace Metal Composites, termed mechanical alloying (MA). Following MA, the mixture was transferred into a steel container and degassed at 773 K, after which the container was sealed airtight under vacuum. Finally, the powder was subjected to hot isostatic pressing (HIP) for 7.2 ks at 1193 K in conjunction with a pressure of 140 MPa to consolidate the material. After HIP, the composite was found to exhibit a heterogeneous microstructure in which the original Ti-3Al-2.5V grains were surrounded with TiB/TiB_2_ at high concentrations. A portion of the TiB_2_ had transitioned to nano-sized needles of TiB, while the remainder was present as large particles of TiB_2_ within zones in which TiB had been formed [36,37,38]. For this reason, additional heating for 14.4 ks at 1473 K in a vacuum tube furnace was used to ensure the complete conversion of the original ceramic to TiB. Subsequent analysis demonstrated that the TiB_2_ particles were completely transformed to micro-sized needles of TiB following this second process [36,37,38]. The resulting composite was extruded at 1273 K while applying a force of 5 MN using a press, in conjunction with a 15:1 extrusion ratio, so as to adjust the orientation of the TiB whiskers. The microstructure of the resulting composite over a region 40,000 μm^2^ in size was characterized by electron probe micro analysis (EPMA) at 500× magnification, operating at a 15 kV acceleration voltage. Furthermore, the microstructure of the composite was characterized using electron backscatter diffraction (EBSD) under the condition of 0.05 µm in a step size at an accelerating voltage of 15 kV to examine the crystal orientation of the TiB whisker.

### 2.2. Experimental Tests

**Figure 1:**
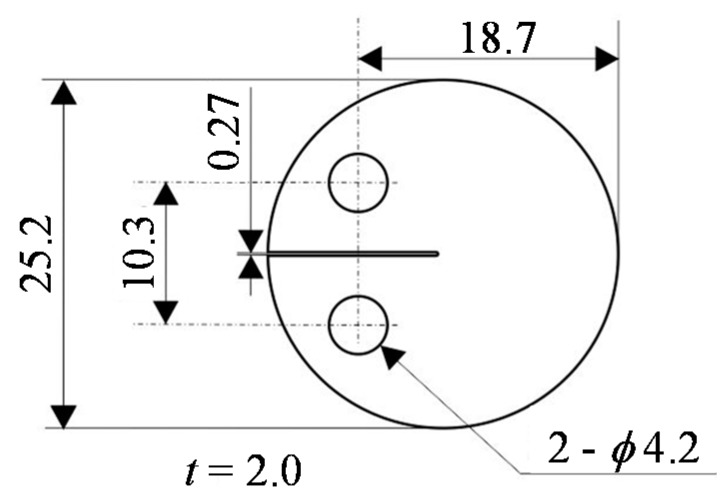
Specimen configuration for fatigue crack propagation tests.

Based on our prior work [39,40,41,42,43,44,45], a disk-shaped compact (DC(T)) sample type was employed in this work, similar to the specimen type referred to in the E399-17 ASTM standard. The composite was machined to produce specimens of this type having the sizes presented in Figure 1, after which the surfaces were polished to a mirror finish using emery papers (#80–#4000) followed by a suspension of SiO_2_. 

**Figure 2:**
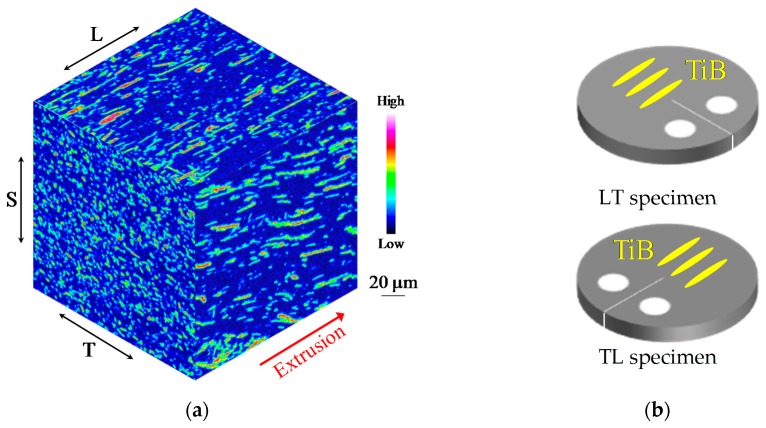
(**a**) Boron map obtained by electron probe micro analysis (EPMA) analysis for TiB/Ti-3Al-2.5V alloy; (**b**) Schematic illustration for the disk-shaped compact (DC(T)) specimens having different TiB orientations.

**Figure 3:**
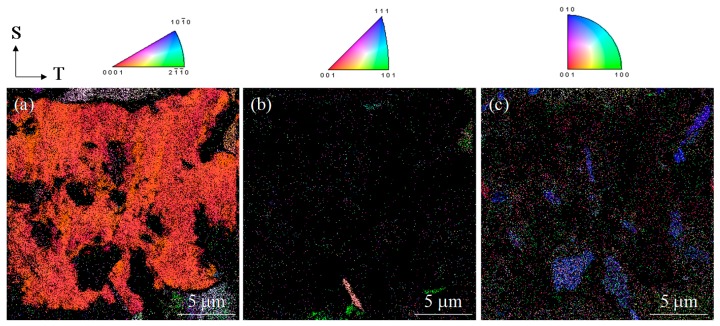
Inverse pole figure maps obtained by electron backscatter diffraction (EBSD) analysis for TiB/Ti-3Al-2.5V composites indicating the crystallographic orientation to the normal plane for (**a**) -Ti, (**b**) -Ti, and (**c**) TiB.

Specimens having two different orientations of TiB whiskers were employed in the present research. Figure 2a provides a map of the TiB/Ti-3Al-2.5V alloy generated by EPMA showing the distribution of boron throughout the material. The positioning of the TiB could be adjusted by varying the direction of the heat extrusion process because the TiB whiskers were automatically oriented parallel to the direction of extrusion. Figure 2b presents a schematic of two DC(T) specimens having different TiB orientations. These were obtained by machining of the bulk composite in the LT plane. In the case of the LT specimens, the TiB whiskers were aligned perpendicular to the crack growth direction, while the TL specimen had whiskers parallel to the crack growth direction. These samples were used to establish the relationship between the TiB orientation and the fatigue crack propagation in the TiB/Ti-3Al-2.5V alloy. Figure 3 shows inverse pole figure (IPF) maps obtained by EBSD analysis for the TiB-reinforced Ti-3Al-2.5V matrix composite at the SL plane. TiB had the same crystallographic orientation represented by blue colors in Figure 3c, and the (010) plane of TiB was perpendicular to the direction of heat extrusion. 

An electrodynamic fatigue test apparatus with the loading capacity of 350 N for DC(T) specimens was used to examine the propagation of fatigue cracks in these specimens, employing three force ratios, *R*, ranging from 0.1 to 0.8. Tests in which the stress intensity factor, *K*, was decreased, were performed in conjunction with a constant *R* value to investigate the fatigue threshold for each sample. In each trial, the specimen was initially pre-cracked to a minimum distance of 1 mm relative to the notch tip, after which stress cycling was carried out at a frequency of 30 Hz under ambient conditions. The threshold stress intensity range, Δ*K*_th_, was taken to equal the largest range of stress intensity values obtained at a crack growth rate of 10^−11^ m/cycle. The unloading elastic compliance technique was used to determine the crack lengths [46]. The extent of crack closure was also estimated by obtaining the closure stress intensity factor, *K*_cl_, from the closure load, *P*_cl_. These data were used to find the effective stress intensity range, Δ*K*_eff_, calculated as *K*_max_ − *K*_cl_, where the former term represents the maximum stress intensity factor (MPa·m^1/2^). Values for *K* were determined using the procedure provided in the E399-17 ASTM standard. The correlation between the backface compliance of the DC(T) specimens and the crack length can be summarized using Equations (1) and (2):

*a/W* = 1 − 2.258*C*_n_ + 0.9048*C*_n_^2^ (1)

*C*_n_*=* 1/[(*ECBW*)^1/2^ + 1] (2)

where *a* represents the length of the crack (m), *W* is the width of the specimen (m), *E* is the Young’s modulus for the sample (GPa), *C* is the compliance, and *B* is the thickness of the specimen (m). Following the crack propagation trials, the crack profiles and fracture surfaces were observed by scanning electron microscopy (SEM). 

## 3. Results and Discussion

### 3.1. Effects of Force Ratio and TiB Orientation on Fatigue Crack Propagation

**Figure 4:**
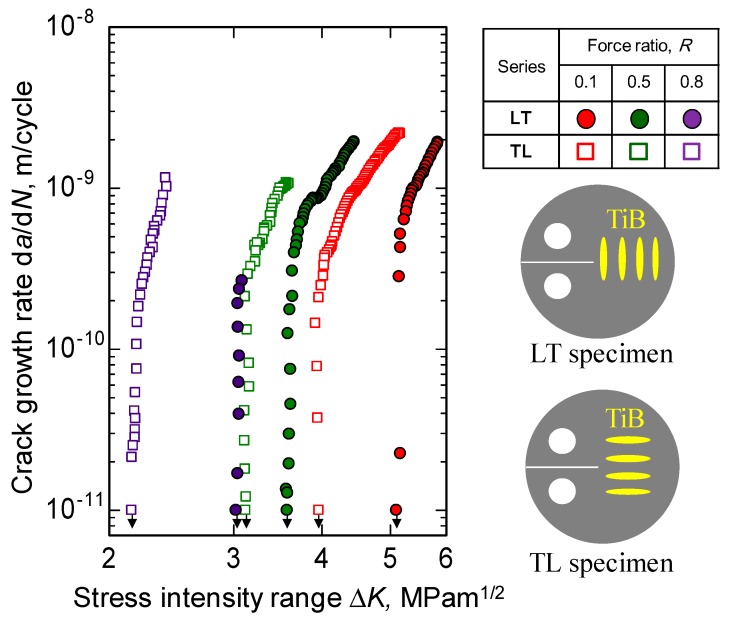
Relationship between crack growth rate and stress intensity range for TiB/Ti-3Al-2.5V composites having different TiB orientations.

The rates of crack growth, d*a*/d*N* for TL and LT specimens are plotted as functions of Δ*K* for *R* values of 0.1, 0.5, and 0.8 in Figure 4. In each data series, Δ*K*_th_ is seen to decrease as *R* is increased while, in contrast, d*a*/d*N* increases. In addition, the values of d*a*/d*N* acquired using the LT specimens (in which the TiB whiskers were perpendicular to the crack growth) are always lower than the values observed using the TL samples at the same Δ*K*. Conversely, the Δ*K*_th_ values of the LT series are always greater than those of the TL series. These findings indicate that fatigue crack propagation in TiB/Ti-3Al-2.5V composites under near-threshold conditions is affected by both the force ratio and the TiB orientation.

### 3.2. Crack Closure

**Figure 5:**
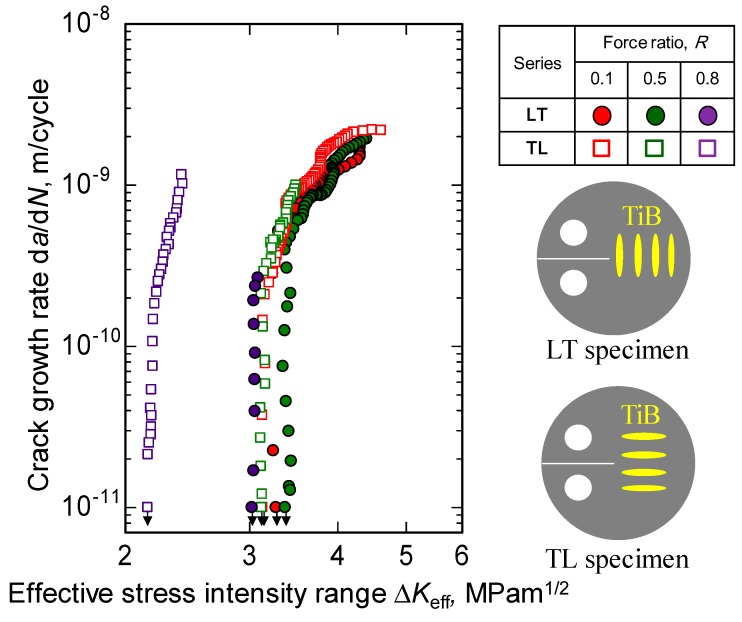
Relationship between crack growth rate and effective stress intensity range for TiB/Ti-3Al-2.5V composites having different TiB orientations.

The effect of the force ratio in conjunction with near-threshold levels is typically ascribed to crack closure [47]. Consequently, this work examined the Δ*K*_eff_ values for TiB/Ti-3Al-2.5V alloys having different TiB orientations. Figure 5 plots d*a*/d*N* as a function of Δ*K*_eff_. For both data series, the data acquired at various force ratios coalesce into a single curve near the threshold at which *R* is less than 0.5. The Δ*K*_eff,th_ values for the LT specimens were very similar, although the Δ*K*_eff,th_ at *R* = 0.8 are slightly lower than those at *R* = 0.1 and 0.5. Boyce and Ritchie [48] have suggested a model based on superposition for investigating the rate of fatigue crack growth in titanium alloys, taking into account cracking due to mechanical fatigue and sustained load at high force ratios. Figure 5 also revealed that the Δ*K*_eff,th_ at *R* = 0.8 for the LT specimens was much higher than that for the TL specimens. The orientation of the TiB whiskers also modifies the sustained load cracking in this alloy.

**Figure 6:**
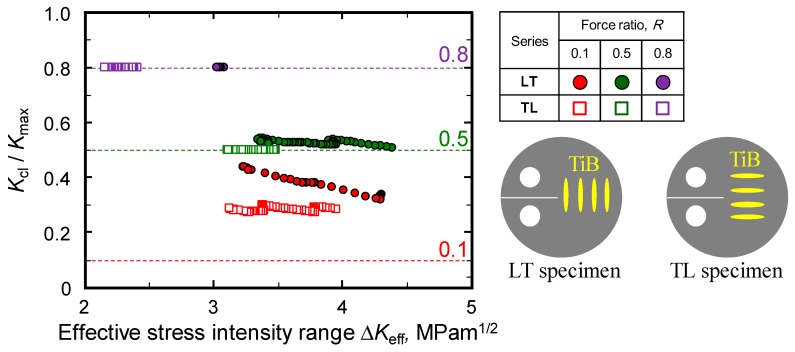
Relationship between *K*_cl_/*K*_max_ and effective stress intensity range for TiB/Ti-3Al-2.5V composites having different TiB orientations.

The effects of both TiB orientation and force ratio on the degree of crack closure were examined by taking the ratio of *K*_cl_, the closure stress intensity factor, to *K*_max_, the maximum stress intensity factor, for each sample. In Figure 6, this ratio is plotted as a function of Δ*K*_eff_ for both orientations at a variety of force ratios. It should be noted that *K*_cl_/*K*_max_ will be equivalent to *R* for a specimen that does not undergo crack closure. The *K*_cl_/*K*_max_ data obtained from both types of sample were clearly not affected by Δ*K*_eff_ at a 0.8 force ratio, demonstrating a lack of crack closure. Conversely, in the case of *R* values less than 0.5, the ratio values obtained from the LT series increased with decreasing Δ*K*_eff_, while the TL series exhibited no crack closure at *R* = 0.5. The LT series also produced a higher *K*_cl_/*K*_max_ than the TL series at *R* of 0.1 and 0.5. It is therefore evident that the orientation of TiB perpendicular to the direction of crack growth promoted crack closure in the alloy, which in turn increased *K*_th_.

### 3.3. Analyses of Crack Profiles and Fracture Surfaces

**Figure 7:**
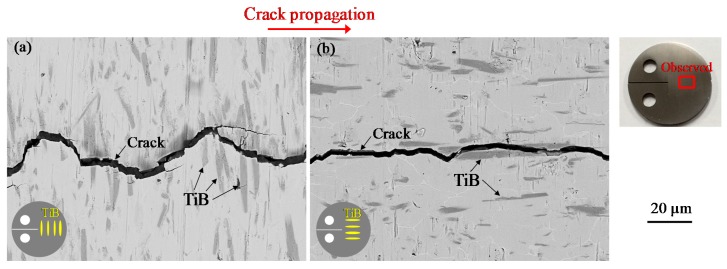
Backscattered electron (BSE) images obtained by SEM observations for crack profiles for (**a**) LT and (**b**) TL series after testing at *R* = 0.1.

The appearance of crack closure in association with near-threshold levels in titanium alloys is commonly ascribed to a roughness-induced process [49,50]. For this reason, the profiles of cracks generated in the present work were observed by SEM. Micrographs of crack profiles in both types of specimens with an *R* of 0.1, obtained using backscattered electron (BSE) imaging, are presented in Figure 7. The contrast in these images reflects the chemical composition of the material. Specifically, brighter contrast is associated with a higher atomic number. Therefore, the TiB showed up as darker than its surroundings. It is apparent that deflected and tortuous cracks were produced in the LT specimens to a greater extent than in the TL series. These results are attributed to the preferential propagation of cracks across boundaries between the TiB and the matrix in certain regions. In the case of the LT specimens, fatigue cracks were also found to propagate through the TiB.

**Figure 8:**
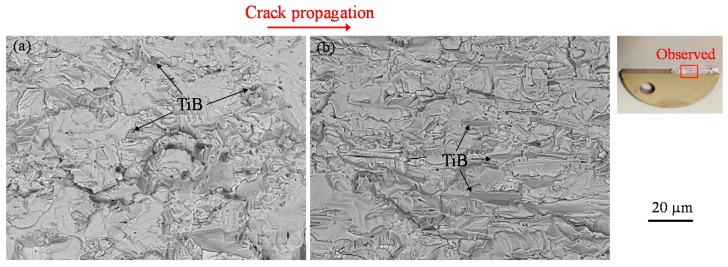
BSE images obtained by SEM observations for fracture surfaces for (**a**) LT and (**b**) TL series after testing at *R* = 0.1.

BSE images of fracture surfaces for both LT and TL specimens following testing with an *R* of 0.1 are provided in Figure 8. In these images, it is clear that TiB was present on the fractured surfaces of both materials. *Ra*, the arithmetic mean deviations for the fracture surfaces, were determined and the values were found to be greater for the LT specimen (11.1 versus 8.65 µm) as a result of the more convoluted crack path in this material. Both the present author [40] and Nalla et al. [51] have previously established that the relationship between structure and fatigue crack growth varies with the alloy microstructure. This effect increases the resistance of the alloy to the growth of fatigue cracks because of the roughness-induced crack closure. The path of cracks in a Ti-3Al-2.5V alloy reinforced with TiB is also affected by the orientation of the TiB. Thus, the fatigue threshold varies with the whisker orientation. For these reasons, deflected and tortuous cracks appeared in the LT specimen, which in turn enhanced the resistance to fatigue crack growth.

## 4. Conclusions

This work examined the effect of the orientation of TiB whiskers on fatigue crack propagation under near-threshold conditions in TiB-reinforced Ti-3Al-2.5V. Surface analyses using both SEM and EPMA identified the fatigue crack propagation mechanism based on the concepts of fractography and crack closure. The primary conclusions that can be drawn from this work are as follows:

1. The orientation of TiB whiskers in a TiB-reinforced Ti-3Al-2.5V alloy determines the crack path;
2. The threshold stress intensity range values, Δ*K*_th_, for a Ti-3Al-2.5V alloy incorporating TiB whiskers oriented perpendicular to the direction of crack growth are greater than that obtained in the case that the orientation is parallel. This effect can be explained by the increase in the degree of the roughness-induced crack closure resulting from the perpendicular TiB, because the deflected and tortuous cracks appear in a Ti-3Al-2.5V alloy reinforced with TiB;
3. The effective threshold stress intensity range values, Δ*K*_eff,th_, determined for the Ti-3Al-2.5V alloy are unaffected by the TiB orientation when the force ratio value is less than 0.5.

## Figures and Tables

**Table 1:** Chemical composition of Ti-3Al-2.5V powder (mass%).

Al	V	Fe	C	O	N	Ti
3.28	2.48	0.04	0.069	0.122	0.008	Bal.

**Table 2:** Chemical composition of TiB_2_ powder (mass%).

B	Fe	C	O	N	Ti
30.0	0.1	0.5	1.1	0.6	Bal.
